# Illegitimate Recombination Between Homeologous Genes in Wheat Genome

**DOI:** 10.3389/fpls.2020.01076

**Published:** 2020-07-21

**Authors:** Chao Liu, Jinpeng Wang, Pengchuan Sun, Jigao Yu, Fanbo Meng, Zhikang Zhang, He Guo, Chendan Wei, Xinyu Li, Shaoqi Shen, Xiyin Wang

**Affiliations:** ^1^School of Life Sciences, North China University of Science and Technology, Tangshan, China; ^2^National Key Laboratory for North China Crop Improvement and Regulation, Hebei Agriculture University, Baoding, China; ^3^Institute for Genomics and Bio-Big-Data, Chengdu University of Traditional Chinese Medicine, Chengdu, China

**Keywords:** common wheat, gene colinearity, gene conversion, polyploidization, homeologous gene

## Abstract

Polyploidies produce a large number of duplicated regions and genes in genomes, which have a long-term impact and stimulate genetic innovation. The high similarity between homeologous chromosomes, forming different subgenomes, or homologous regions after genome repatterning, may permit illegitimate DNA recombination. Here, based on gene colinearity, we aligned the (sub)genomes of common wheat (*Triticum aestivum*, AABBDD genotype) and its relatives, including *Triticum urartu* (AA), *Aegilops tauschii* (DD), and *T. turgidum* ssp. dicoccoides (AABB) to detect the homeologous (paralogous or orthologous) colinear genes within and between (sub)genomes. Besides, we inferred more ancient paralogous regions produced by a much ancient grass-common tetraploidization. By comparing the sequence similarity between paralogous and orthologous genes, we assumed abnormality in the topology of constructed gene trees, which could be explained by gene conversion as a result of illegitimate recombination. We found large numbers of inferred converted genes (>2,000 gene pairs) suggested long-lasting genome instability of the hexaploid plant, and preferential donor roles by DD genes. Though illegitimate recombination was much restricted, duplicated genes produced by an ancient whole-genome duplication, which occurred millions of years ago, also showed evidence of likely gene conversion. As to biological function, we found that ~40% catalytic genes in colinearity, including those involved in starch biosynthesis, were likely affected by gene conversion. The present study will contribute to understanding the functional and structural innovation of the common wheat genome.

## Highlights

Homeologous genes in common wheat, likely converted by one another, show long-lasting genome instability after polyploidization.

## Introduction

Common wheat is one of the most widely grown cereal crops in the world and an essential source of food, its production affects the global economy, and failed harvests could lead to social unrest (Choulet et al., 2014; Wang et al., 2015; Appels et al., 2018). The sequencing of its relatives, the diploid *Triticum urartu* (genotype A_d_A_d_; hereafter capital letters to indicate genome types and subscripts to indicate ploidy of organisms carrying them, with d, t, or h to show diploid, tetraploid, or hexaploid, respectively), and *Aegilops tauschii* (genome D_d_D_d_), and the tetraploid wild emmer wheat (*T. turgidum* ssp. dicoccoides, genome A_t_A_t_B_t_B_t_) contributed to understanding their genome evolution and innovation (Middleton et al., 2014; Du et al., 2017; Zimin et al., 2017; Gardiner et al., 2019). Wheat, as a heterologous hexaploid (genotype A_h_A_h_B_h_B_h_D_h_D_h_), was possibly produced when *T. urartu* hybridized with an ear of wheat carrying a B genome, thereby forming a neo-tetraploid that hybridized with the *A. tauschii*. Two hybridization process made the common wheat genome complicated and made its genome even more complicated is that a grass-common whole-genome duplication (cWGD) having occurred ~100 million years ago produced thousands of duplicated genes in the extant grass genomes (Paterson et al., 2004; Wang et al., 2015; Du et al., 2017). The event played an important role in promoting the formation of new species of grasses (Sun et al., 2017).

Genetic recombination is the primary source of genetic novelty (Kurosawa and Ohta, 2011). In plants, meiotic and mitotic recombinations are reciprocal, involving asymmetric exchange of genetic information between the homologs (Jia et al., 2013; Gardiner et al., 2019). Whereas, irreversible recombination involves the transfer of information from one site to its homeolog, leading to gene transformation (Datta et al., 1997; Kurosawa and Ohta, 2011). Gene conversion involves the unidirectional transfer of genetic material from a ‘donor’ sequence to a homologous ‘acceptor.’ In eukaryotes, it constitutes the main form of homologous recombination initiated by DNA double-strand breaks, as reviewed previously (Chen et al., 2007).

Whole-genome duplication will result in a large number of paralogous genes on homeologous chromosomes or chromosomal regions, and substantial similarity between homeologous regions could permit the occurrence of illegitimate recombination (as compared to normal homologous recombination), often resulting in a pattern called gene conversion that could be explained by that a gene is replaced by its homologous genes (Wang et al., 2009). There were reports to show that gene conversion could have occurred between paralogs in grasses and other plants. In some cases, gene conversion could have been frequent and is even on-going between paralogs on the homeologous chromosomes, e.g., rice chromosome 11 and its cWGD homeolog, chromosome 12 (Wang et al., 2009; Kurosawa and Ohta, 2011; Wang et al., 2011; Wang et al., 2019).

Illegitimate recombination has profound consequences on the evolution of paralogous genes and the chromosomes that they reside in. While paralogous recombination elevates base mutation rates, evolutionary rate, and DNA sequence deletion, converted regions have highly similar paralogous DNA (Wang et al., 2009; Wang et al., 2011). This is a seemingly perplexing but an essence reasonable finding. Temporally segmental restriction of paralogous recombination along homeologous chromosomes has produced stepwise strata of increased similarity from their centromeres to chromosome ends (Wang and Paterson, 2011). Increased mutation rates may foster genetic innovation. A significant QTL (qS12) resulting in hybrid male sterility was mapped within ~400 kb region adjacent to the highly recombined areas on the short arm of chromosome 12 (Zhang et al., 2011). Moreover, two genes harboring the E3-ligase RING-C2 domain on the distal parts of rice chromosomes 11 and 12, were identified, and have possibly evolved in concert *via* gene conversion (Jung et al., 2012).

Despite thousands of paralogs in wheat genomes produced by recurrent polyploidies, a comprehensive analysis of gene conversion is lacking. Here, by aligning the wheat and its relatives’ genomes, we inferred gene colinearity within a genome and orthology between them, and eventually characterized the pattern of illegitimate recombination and gene conversion. The present work will contribute to the understanding of genetic and structural innovation of wheat genes and chromosomes.

## Results

### Alignment of Wheat and Relative Genomes

Chromosome homeology was revealed by inferring colinear genes using the software ColinearScan (Wang et al., 2006). We revealed 232 A_h_B_h_, 187 A_h_D_h_, and 154 B_h_D_h_ homeologous regions between wheat subgenomes, involving 14,161, 15,376, and 15,553 homeologous genes, respectively (**Table 1**). This meant about 40% of their genes were involved in inter-subgenome colinearity. Actually, as to the numbers and percentages of colinear genes between genomes, B_h_D_h_ shared better gene colinearity than the other pairs of subgenomes; while A_h_B_h_ shared the least gene colinearity. As to each of the three subgenomes, D_h_ shared a better colinearity with the other two subgenomes. This seems to agree with the inference that D_h_ subgenome joined the latest to produce the hexaploid wheat and/or has a conservative nature, as discussed later.

**Table 1:** The Number of colinear genes within and among the wheat and relative genomes.

Species	Colinear block#	Colinear gene#	Percent 1 (%)	Percent 2 (%)
D_d_A_h_	305	17,157	44.25	47.26
D_d_B_h_	336	17,373	44.80	47.29
D_d_D_h_	374	20,474	52.80	58.46
D_d_A_d_	196	9,886	25.50	26.31
D_d_A_t_	175	16,848	43.45	54.83
D_d_B_t_	278	16,030	41.34	49.96
A_h_A_h_	56	2,673	7.36	7.36
A_h_B_t_	236	12,954	35.68	40.38
B_h_B_h_	43	2,139	5.82	5.82
D_h_D_h_	41	2,185	6.24	6.24
A_d_A_h_	211	8,195	21.81	22.57
A_d_B_t_	178	7,361	19.59	22.94
A_d_B_h_	183	7,886	20.99	21.47
A_d_D_h_	191	8,507	22.64	24.29
B_t_B_h_	97	13,913	43.37	37.87
B_t_D_h_	244	14,093	43.93	40.24
A_h_B_h_	232	14,161	39.01	38.55
A_h_D_h_	187	15,376	42.36	43.91
B_h_D_h_	154	15,553	42.33	44.41

B_t_, subgenome B in tetraploid wild wheat; A_h_, subgenome A in common wheat; B_h_, subgenome B in common wheat; D_h_, subgenome D in common wheat; A_d_, Triticum urartu; D_d_, Aegilops tauschii. Percent 1 and 2, Percentages of colinear genes accounting total genes in the first and second (sub)genomes shown in the first column.

**Figure 1:**
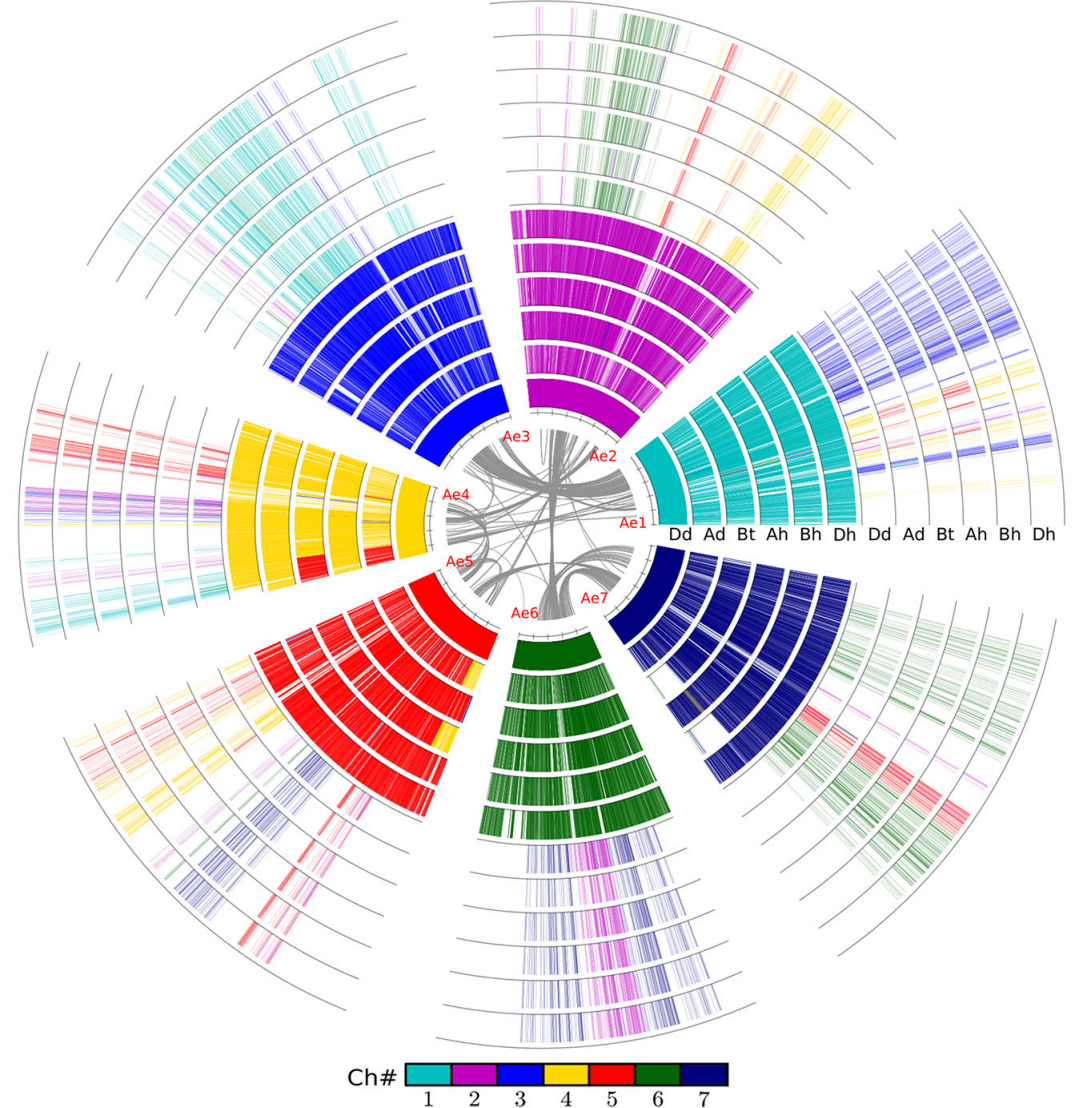
Alignment of the wheat and relative genomes with wheat DD as reference. The whole-genome duplication (WGD) in the common grass ancestor of plants caused them to have at least two circles of chromosomes. The hybridization event caused common wheat to have six such chromosomes. The innermost circle represents the seven chromosomes of the wheat DD genome (D_d_) from *Aegilops tauschii*, and the gray lines linking paralogous genes. A_d_, *Triticum urartu*; B_t_, subgenome B in tetraploid wild wheat; A_h_, B_h_, and D_h_, three subgenomes of common wheat.

Similarly, we detected orthologous regions and genes between the diploid wheat relative genomes (A_d_A_d_ from *T. urantu* and D_d_D_d_ from *A. tauschii*) and the corresponding common wheat subgenomes. Actually, with the same parameters, we inferred 211 A_d_A_h_ and 374 D_d_D_h_ orthologous regions, including 8,195 and 20,474 colinear genes, respectively. Notably, the D_d_ and D_h_ shared much better orthology than the A_d_ and A_h_ did, possibly due to much more genome fractionation in the A_d_ and A_h_. This fact also supports D_h_’s being a later player to form the hexaploid. We also revealed gene colinearity between the tetraploid wild wheat, *T. turgidum* (A_t_A_t_B_t_B_t_), with the common wheat genome. Mainly, the tetraploid B_t_ subgenome and hexaploid B_h_ subgenome shared 97 orthologous regions, involving 13,913 colinear genes and 80.0% of these colinear genes are supported by OrthorMCL (**Table 2**, **Figure 1**).

**Table 2:** Number of paralogous and orthologous genes supported by OrthoMCL within and among wheat and relative genomes.

Species	Colinear gene#	Colinear gene in OrthoMCL#	Percent (%)
D_d_A_h_	17,157	13,825	80.58
D_d_B_h_	17,373	13,412	77.20
D_d_D_h_	20,474	17,126	83.65
D_d_A_d_	9,886	8,557	86.56
D_d_A_t_	16,848	13,421	79.66
D_d_B_t_	16,030	13,321	83.10
A_h_A_h_	2,673	2,171	81.22
A_h_B_t_	12,954	10,210	78.82
B_h_B_h_	2,139	1,701	79.52
D_h_D_h_	2,185	1,738	79.54
A_d_A_h_	8,195	6,551	79.94
A_d_B_t_	7,361	6,023	81.82
A_d_B_h_	7,886	6,548	83.03
A_d_D_h_	8,507	6,849	80.51
B_t_B_h_	13,913	11,131	80.00
A_h_B_h_	14,161	11,512	81.29
A_h_D_h_	15,376	12,305	80.03
B_h_D_h_	15,553	12,105	77.83

Percent, Percentage of colinear genes supported by OrthoMCL results.

### Gene Conversion Between Common Wheat Subgenomes

**Figure 2:**
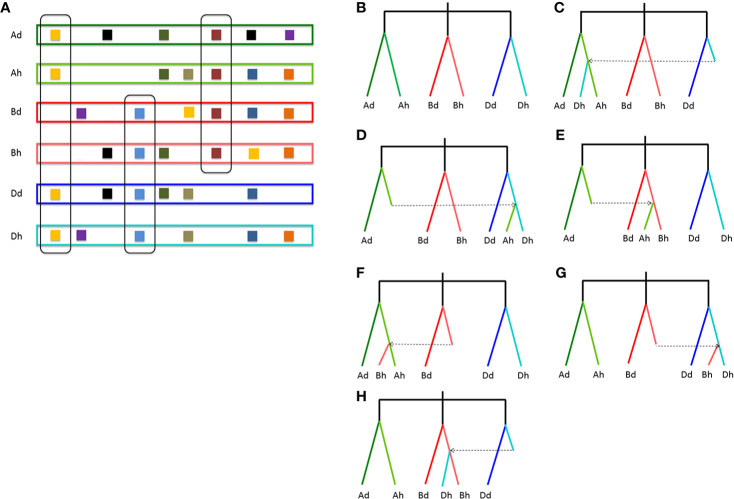
Homologous gene quartets and inference of conversion based on phylogenetic topology changes. **(A)** Colinear genes and quartets of homologous genes. Squares show genes, and the same color ones show homologous genes. If conversion does not occur, the expected phylogenetic relationship of a homologous quartet is shown in **(B)**; **(C)** if a D_h_ gene is converted by an A_h_ paralog; **(D)** if an A_h_ gene is converted by a D_h_ paralog; **(E)** if an Ah gene is converted by a B_h_ paralog; **(F)** if a B_h_ gene is converted by an A_h_ paralog; **(G)** if a B_h_ gene is converted by a D_h_ paralog; **(H)** if a D_h_ gene is converted by a B_h_ paralog. Places without squares indicate gene loss.

To infer likely gene conversion, we retrieved homologous gene quartets from the above constructed multiple alignments. With D_d_ as the reference, we assumed 7,462 A_d_A_h_D_d_D_h_ homeologous gene quartets, with each quartet containing a D_d_ gene, and its ortholog in common wheat D_h_ subgenome, and their corresponding orthologs in A_d_ genome and common wheat A_h_ genome (**Table 1**). This meant that about 42.36–43.91% of colinear genes in each genome were involved in the revealed quartets (**Figure 2A**). Theoretically, the common wheat A_h_D_h_ homeologous genes were more diverged than each with their diploid orthologs. However, due to gene conversion following illegitimate recombination, the homeologs might be more similar to one another, resulting in an aberrant tree topology. The phylogenetic tree of the homologous quartet with gene conversion is shown in **Figures 2C–H**. By checking tree topology after aligning the gene sequence of each quartet, we found that 164 common wheat homeologs (~2.20% of all those involved in quartets) were likely converted.

**Figure 3:**
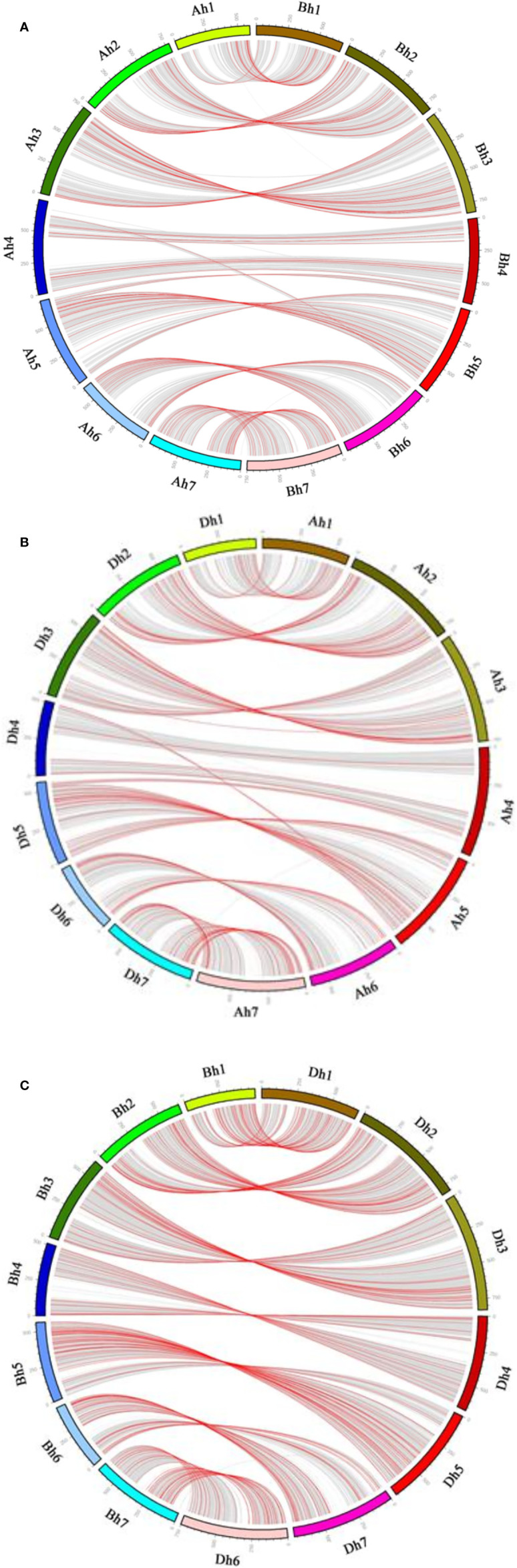
Gene conversion between wheat subgenomes. In each subfigure, the outer circle shows the seven chromosomes of each of wheat subgenome. Non-converted homeologous gene pairs between the subgenomes are connected by curvy gray lines, converted pairs by curvy red lines. **(A)** A_h_-B_h_; **(B)** A_h_-D_h_; **(C)** B_h_-D_h_.

For there was not a diploid B genome available, we took the B_t_ genome from the emmer wheat in the analysis and constructed A_d_A_h_B_t_B_h_ and B_t_B_h_D_d_D_h_ quartets (**Table 3**). We inferred 6059 A_d_A_h_B_t_B_h_ quartets and found that 127 (or 2.10% of all those involved in quartets) A_h_B_h_ homeologs were likely converted, showing much more conversion than the B_h_D_h_ homeologs. Similarly, we inferred 12,770 B_h_–D_h_ quartets, and found that 253 (or 1.99% of all those involved in quartets) were likely converted, showing similar gene conversion rates between subgenomes (**Figure 3**). With the converted homeologs, one copy might act as the donor, while the other as the acceptor. If one gene was taken as the donor, the sequence of the acceptor would be converted to be identical or highly similar, immediately after the conversion, to the donor’s ortholog in the diploid (tetraploid) plant. It seemed that in A_h_D_h_ gene conversions, about 62.20% of the converted genes took the D_h_ homeologs as the donor. This showed that the subgenome D was more likely taken as the repairing template during recombination with the subgenome A. With A_h_B_h_ and B_h_D_h_ converted genes, the B_h_ homeologs were more often (59.84 and 66.40%) taken as the donors, respectively, showing the subgenome B was often taken as the repairing template when pairing with the subgenomes A or D (**Table 3**).

**Table 3:** Gene conversion between subgenomes in common wheat.

Comparisons	Quartet	Gene conversion	Percent1 (%)	Donor	Percent2(%)
A_d_A_h_B_t_B_h_	6,059	127	2.10	51/76	40.16/59.84
A_d_A_h_D_d_D_h_	7,462	164	2.20	62/102	37.80/62.20
B_t_B_h_D_d_D_h_	12,770	253	1.99	168/85	66.40/33.60

See the note in **Table 1**. Percent1: Percentage of gene conversion occupies the proportion of quartet. Percent2: Percentage of donor gene occupies the portion of gene conversion. Donor: Genes that provide homologous genes during gene conversion.

### The Lower Gene Conversion Rate Between Ancient Paralogs Produced by the Grass Common Whole-Genome Duplication

We found likely gene conversion between ancient paralogs in each subgenome produced by the cWGD ~100 million years ago. To see gene conversions between ancient paralogs, such as those in the subgenome A_h_, we first revealed the paralogs having preserved colineartiy resulting from the cWGD, inferred their A_d_ orthologs, and found the A_d_A_h_A_d_A_h_ quartets. Then, we checked likely tree topology changes, being aberrant from the expected one. The orthologs were supposed to be more similar than each to the paralog in the same subgenome. However, if the paralogs, such as those A_h_ paralogs, were more similar, it showed likely gene conversion. Here, we found about 700 homologous quartets within each of the three subgenomes and showed that ancient gene conversion rates varied from 0.83 to 1.71% (**Table 4**), being lower (though not significantly) than those between these subgenomes, as estimated above.

**Table 4:** Gene conversion between paralogs in common wheat.

Comparisons	Quartet	Gene conversion	Percent (%)
A_d_A_h_A_d_A_h_	700	12	1.71
B_t_B_h_B_t_B_h_	721	8	1.11
D_d_D_h_D_d_D_h_	723	6	0.83

### Gene Function Analysis

**Figure 4:**
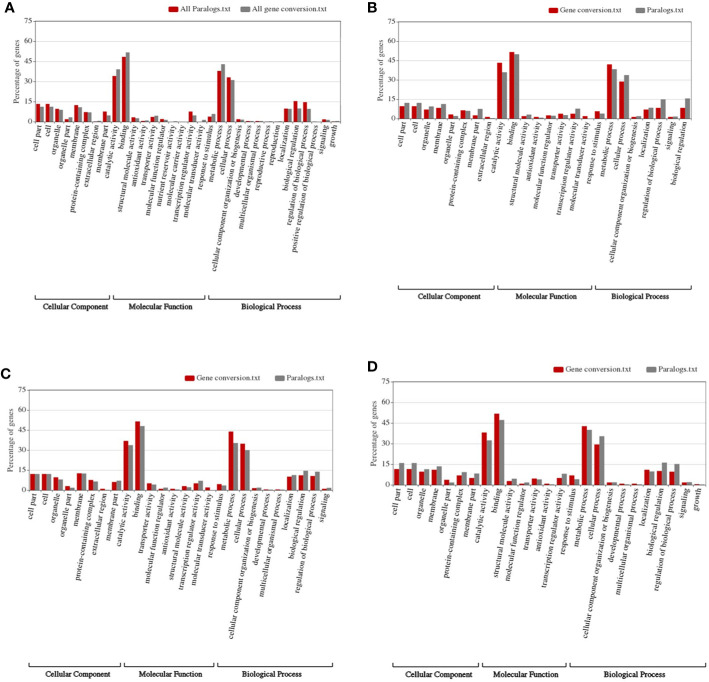
Construction of functional spectrum of subgenomes in wheat. **(A)** whole genome; **(B)** Ah subgenome; **(C)** Bh subgenome; **(D)** Dh subgenome.

By performing Gene Ontology analysis, we found that, in the whole genome level, specific functional genes were enriched (**Figure 4**). The organelle-related functional genes were significantly more likely to be converted compared to all colinear genes (~3.32 to 1.89%, Fisher′s exact test p-value = 0.041). Similarly, the organelle extracellular region genes were significantly more likely to be converted compared to all colinear genes (~0.70% to 0, p-value = 0.004). Genes involved in the nutrient reservoir and molecular transduction were also significantly likely affected by conversion (**Figure 4D**). Comparatively, the membrane part genes and biological regulation genes showed substantially less likely to be involved in conversion as compared to all colinear genes (~4.71 and 7.63%, p-value = 0.0182; and ~9.95 and 15.45%, p-value = 0.002) (**Figure 4A**).

As to each subgenome, specific genes were enriched in converted genes (**Figures 4B–D**). For subgenome B, the molecular transducer activity genes showed a significant increase in converted genes (~0 to 2.02%, p-value = 0.0004). As to the signaling genes in subgenome A, those in subgenome D showed a considerable increase (1.27 and 10.09%, p-value = 0.0002). The biological regulation genes in subgenome D showed a significant increase (~1.83 to 8.28%, p-value = 0.003).

**Figure 5:**
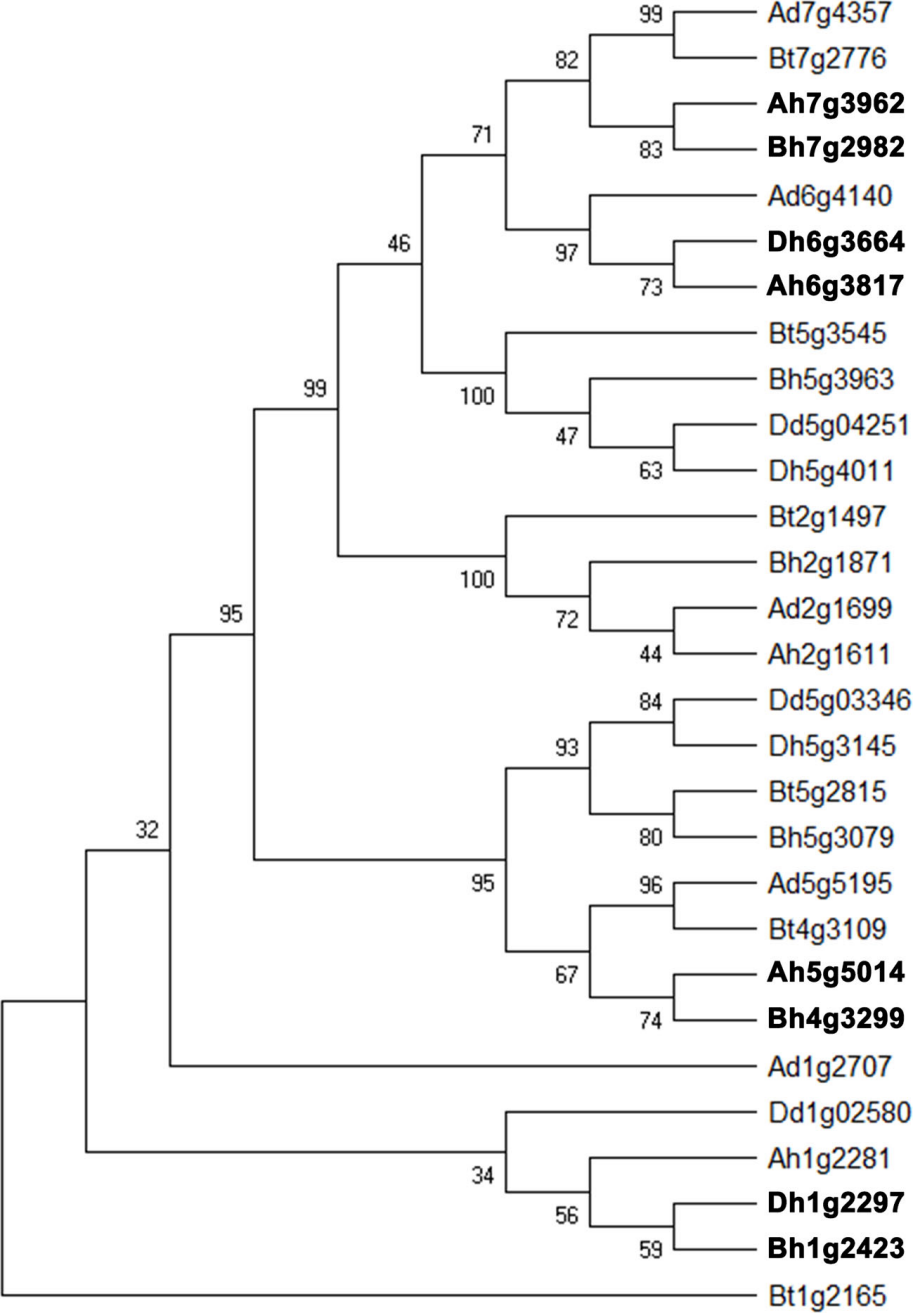
Phylogenetic tree of homologous gene quartet with catalytic activity in starch biosynthesis. B_t_, subgenome B in tetraploid wild wheat; D_d_, *Aegilops tauschii*; A_d_, *Triticum urartu*; A_h_, B_h_, and D_h_, three subgenomes of common wheat. Gene names in bold font show conversion affected ones.

Starch, one of the final products of photosynthesis, helps form the wheat seeds. About 65–80% of the grain composition of common wheat is starch. Here, we found that about 40% of starch biosynthesis catalytic genes were likely involved in gene conversion in the whole genome and each its subgenome. Therefore, we selected a set of catalytic genes regulating starch biosynthesis, the converted ones, and their close homologs in wheat and relative (sub)genomes to construct a phylogenetic tree (**Figure 5**). The converted genes shown here are Ah7g3962/Bh7g2982, Ah6g3817/Dh6g3664, Ah5g5014/Ah4g3299, Bh1g2423/Dh1g2297, each pair showing conversion between different subgenomes.

## Discussion

The doubling of the whole-genome often leads to an extremely unstable genome of the species (Freeling et al., 2012; Zhang et al., 2013; Wang et al., 2018). Illegitimate recombination, in contrast to recombination usually between homologous chromosomes, may occur between homeologous chromosomes (Wang et al., 2009; Woodhouse et al., 2010; Murat et al., 2014). Genome instability is often characterized by large-scale DNA losses, DNA rearrangement, even chromosome breakages, and fusions, which eventually contribute to regaining genome stability. After some time, which is difficult to define how long it is, the duplicated genome would come to a calm state. However, though at a much-lowered rate, illegitimate recombination may still occur between homeologous chromosomal regions. In rice, sorghum and other grasses, we found illegitimate recombination could last tens of millions of years, resulting in a particular stratum structure between two rice homeologous chromosomes, and between their sorghum counterparts, and many involved rice and sorghum genes might have been converted by their homeologous copies (Wang et al., 2009; Wang et al., 2011).

Here, we checked likely gene conversion in wheat, an important world-wide crop, and found hundreds of converted genes between the subgenomes. This shows that the wheat genome has not been quite stable after the formation of its hexaploid structure about 10,000 years ago (Feldman et al., 2012). There has been genetically illegitimate recombination between the subgenomes, contributing to the structural and functional changes of homeologous chromosomes and the genes residing on them. We also found likely gene conversion between more ancient duplicated genes produced by the cWGD having occurred ~100 million of years ago (Wang et al., 2015). Though the conversion scale of duplicated genes within each subgenome is lower than that between different subgenomes, it suggested the long-lasting lingering effect of illegitimate recombination after the whole-genome duplication, as reported previously in rice and its *Oryza* relatives, sorghum, and other grasses (Jacquemin et al., 2009; Wang et al., 2011; Ling et al., 2013).

Notably, we revealed that genes from different common wheat subgenomes tend to play different roles during the occurrence of conversion. The subgenome DD shares more colinear genes with the other two subgenomes and its genes act more likely as donors, showing likely preferential roles of DD chromosomes and the genes residing on them. This may be related to the fact that subgenome DD was the last one to be added to form the hexaploid plant, common wheat (Middleton et al., 2014; Du et al., 2017; Zimin et al., 2017; Gardiner et al., 2019). The initial formation of a tetraploid ancestor (AABB) may have been followed likely wide-spread DNA and gene losses, accompanying illegitimate recombination between homeologous chromosomes, due to genomic instability of the tetraploid. After this turmoil stage, the homeologous chromosomes became much different, and the tetraploid genome came to a relatively stable state. Then the third genome DD, contributed by an ancient plant that *A. tauschii* is derived from, hybridized with the tetraploid to form the hexaploid common wheat. The newly joined subgenome DD lives much at ease with the reduced DNA content of the subgenomes AA and BB. Though there should have been continual DNA losses and illegitimate recombination, the occurrence rates should have likely decreased. This eventually resulted in a better gene colinearity of the subgenome DD with the other two than the other ones with one another, as observed above. Furthermore, relatively innocent state of DD genes might render themselves privileges to act as the donors during gene conversion, to preserve ancestral gene structures and functions.

The above-inferred conversion involved homeologs produced by the formation of the hexaploid about 10,000 years ago, while the tetraploid progenitor (AABB) might have formed at least 0.5 million years ago (Marcussen et al., 2014; Brenchley et al., 2012). Comparatively, the divergence of their diploid ancestor occurred at least 2.5 to 7 million years ago (Marcussen et al., 2014; Cheng et al., 2019). With updated fossil evidence applying to colinear genes, a grass-family level estimation of their Triticeae common ancestor suggested its appearing more than 20 million years ago (Wang et al., 2015). These facts mean that the wheat homeologs have much diverged from one another and phylogenetic inference of gene conversion between homeologs by comparing their close orthologs in diploid or tetraploid progenitor would not likely result in high false positives. This supports the credibility of the present gene conversion inference. However, this could really be an underestimation of gene conversion. Thus, it is not easy of converted paralogs to be well supported at such rigorous criteria about gene tree topology change. The subgenome copies are quite similar to each other, and there may be no much time to gather mutations between the diploid-hexaploid orthologs to show likely occurrence of gene conversion between the hexaploid subgenome copies.

Notably, we found that around 40% of starch biosynthesis catalytic genes were involved in gene conversion, showing that conversion may favor certain types of functional genes, as shown with the above pilot characterization with GO. Actually, while characterizing the phylogeny of those catalytic genes, the constructed phylogenetic tree involved low Bootstrap values in some branches. However, the credibility of the tree was well supported by known or expected phylogenetic relationship, paralogy between homeologs from subgenomes, or orthology between homeologs from different organisms. Actually, the known or expected relationship between homeologs was further supported by inferred gene colinearity, which is a good criteria to explore the real phylogeny, especially when it was disturbed by non-uniform evolutionary rates of plant genes (Meng et al., 2020).

## Materials and Methods

### Materials

The genome-wide datasets, including whole-genome sequences, annotated genes, and their translated proteins, were retrieved from following public databases.

*Tritium aestivum*: URGI, https://wheat-urgi.versailles.inra.fr; *Triticum urartu*: MBK, http://www.mbkbase.org; *T.turgidum* ssp.dicoccoides: http://wewseq.wixsite.com; *Aegilops tauschii*: http://aegilops.wheat.ucdavis.edu/ATGSP.

### Gene Colinearity Inference

By using the similarity comparison tool BLASTP compared the proteins within and between the genomes of wheat and related species, and scoring according to the similarity between the protein sequences, we inferred the relationship of gene homology according to the results of the comparison, and the expected value (E-value) is limited to 1e−5. Then the result of BLASTP was used for the software ColinearScan to infer colinear gene pairs. Colinear genes are those in a genomic region sharing locational orders with their homologs in another genomic region. The detection of genomic homologous colinear fragments was based on the principle of similarity between genes, which was combined with the position information of genes on the chromosomes. The colinearity analysis software ColinearScan was used to infer homologous regions (or blocks) containing five or more colinear genes (Wang et al., 2017). The maximal searching gap between colinear genes was set to be 50 intervening non-colinear genes(P <0.05), as often adopted in previous analyses (Sun et al., 2017). Besides, we used OrthoMCL (http://www.micans.org/mcl/src/mcl-latest.tar.gz) to identify orthologous and paralogous genes to support the credibility of the gene colinearity inferred above (Li et al., 2003; Brenchley et al., 2012).

### Inference of Homologous Quartets and Gene Conversion

We used wheat *A. tauschii* genomes as a reference to construct multiple alignments. In details, each genome was compared and aligned to the referenced genome by inferring gene colinearity; any two genomes were compared to find inter-genome colinearity; and then, each genome was aligned to itself to find paralogous genes produced by cWGD (Wang et al., 2015; Ling et al., 2018; Liu et al., 2019). We used multiple alignments to construct homologous gene quartets based on the homologous relationships. By checking colinear genes in the multiple alignments, the paralogous gene pairs in each of the involved genomes and the orthologous gene pairs between genomes were obtained, and homologous gene quartets were retrieved. Using these quartets, we inferred gene conversion based on the aberrant topology of each homologous gene quartet. Aberrant tree topology is that the relationship between homeologous genes is different from the expected phylogeny (**Figure 2A**). With each gene quartet, multiple sequence alignment was performed using ClustalW (Thompson et al., 1994). If in the alignment, the number of gaped sites in any of the aligned sequences is more than 50% of the alignment length or the amino acid identity was less than 40%. Then the alignment was discarded for further inference (Wang et al., 2009) As to the conversion inference with each homologous quartet, phylogenetic gene trees were constructed by using Neighbor-joining approach implemented in MEGAX with encoded amino acid sequences as input, and default parameters were adopted (Tamura et al., 2007). Bootstrap of 1,000 iterations was performed to improve the reliability of the evolutionary tree. Then, with trees supported by bootstrap value >70%, we checked the tree topology to find whether there was likely gene conversion. Synonymous nucleotide substitutions on synonymous substitution sites (Ks) between homologous genes (Nei and Gojobori, 1986) were estimated to characterize the distance between homologs with the program implemented in Bioperl (Stajich et al., 2002). Whole-genome scale gene conversion was displayed by using Circos software (Krzywinski et al., 2009).

### GO Enrichment Analysis

We performed GO enrichment analysis and used the domain similarity comparison software InterProScan to annotate genes with GO (Gaudet, 2019). The best international standard matching wheat genes was obtained through the BLASTP comparison between the international standard protein database and the whole wheat genomic protein file. Then, we used WEGO2.0 (http://wego.genomics.org.cn/) to generate the wheat gene function map. The log function standardized the number of genes involved in each category, and then the functional distribution and correlation were analyzed. The p-value was calculated using hypergeometric delivery to explain the significance of gene enrichment. Here we selected a set of starch catalytic activity function genes to build a phylogenetic tree. CLUSTALW was used to perform sequence alignment on the protein sequences (Thompson et al., 1994), and a phylogenetic tree was constructed using the Maximum Likelihood (Murshudov et al., 1997). The pilot value was based on 1,000 copies.

## Data Availability Statement

The datasets generated for this study can be found in the URGI, https://wheat-urgi.versailles.inra.fr.

## Author Contributions

XW conceived and led the research. CL, JW, PS, JY, FM, ZZ, HG, CW, and XL performed the analysis or contributed analysis tools. XW and CL wrote the paper. All authors contributed to the article and approved the submitted version.

## Conflict of Interest

The authors declare that the research was conducted in the absence of any commercial or financial relationships that could be construed as a potential conflict of interest.

The handling editor declared a past co-authorship with one of the authors XW.
