# Supplementary material for: Illegitimate Recombination Between Homeologous Genes in Wheat Genome
Source: Front Plant Sci. 2020 Jul 21;11:1076. doi: 10.3389/fpls.2020.01076 (PMC7396543; doi:10.3389/fpls.2020.01076)
Supplement: Supplementary file 1 [file Table_1.docx]

SUPPLEMENTARY MATERIAL

Table 1 Information of genomes.

| **Species name** | **Common name** | **Genes on chr** | **Data source** |
| --- | --- | --- | --- |
| *Oryza sativa* | Rice | 38714 | MBK  (https://www.mbkbase.org) |
| *Aegilops tauschii* | Wheat D | 38775 | ATGSP  (http://aegilops.wheat.ucdavis.edu/ATGSP/annotation/) |
| *Triticum aestivum* | Common wheat | Genome A 36302 | URGI  (https://wheat-urgi.versailles.inra.fr/) |
|  |  | Genome B 36738 |  |
|  |  | Genome D 35021 |  |
| *Triticum urartu* | Wheat A | 37576 | MBK  (http://www.mbkbase.org/Tu) |
| *T. turgidum ssp.*  *Dicoccoides* | Tetraploid wild wheat | Genome A 30730 | http://wewseq.wixsite.com/consortium. |
|  |  | Genome B 32083 |  |
